# Case Report: Possible improvement of near-adult height with PEG-rhGH and letrozole in a late-puberty boy with atypical Noonan syndrome and bone age/height age discrepancy: a novel therapy

**DOI:** 10.3389/fped.2026.1643512

**Published:** 2026-04-13

**Authors:** Zixia Zhang, Miaomiao Li, Xue Wu, Xi Wang, Jiaqian Hu, Mengqin Wang, Linfei Li, Yongxing Chen

**Affiliations:** 1 Department of Endocrinology and Genetics and Metabolism, Children's Hospital Affiliated to Zhengzhou University, Henan Children's Hospital Zhengzhou Children's Hospital, Zhengzhou, Henan, China; 2 Henan Pediatric Clinical Research Center, Zhengzhou, Henan, China

**Keywords:** case report, letrozole, Noonan syndrome, pegylated recombinant human growth hormone, short stature

## Abstract

**Background:**

Noonan syndrome (NS) is a rare congenital disorder predominantly characterized by short stature, with recombinant human growth hormone (rhGH) as the primary treatment. This study aimed to investigate the effects of a novel therapy involving pegylated (PEG)-rhGH and letrozole on near-adult height (NAH) improvement in a boy with NS who was undergoing late-puberty and presented with higher bone age (BA) than height age (HA).

**Case presentation:**

We reported a 13-year-old boy with NS presented with subtle NS facial features, notable short stature and a higher BA than HA. His predicted adult height (PAH) was 163.3 cm (genetic target height: 178 cm). Following a therapeutic regimen of PEG-rhGH (2.3 years) and letrozole (1.7 years), his height increased by 9.7 cm in the first year, achieving an average growth rate of approximately 8.04 cm over a period of 2.3 years. The BA minus chronological age (CA) decreased from −0.33 to −1.50, indicating that letrozole slowed BA progression and prolonged the growth period. Ultimately, his NAH reached 172.1 cm, the PAH increased by 8.8 cm, and the height standard deviation score based on CA (HtSDS _CA_) improved by 1.62. Additionally, there were no significant changes in thyroid function, blood glucose, uric acid, and IGF-1 levels during the treatment period, which fluctuated within the normal range. The patient had a transient slight elevation in testosterone, but normalized spontaneously, and the patient reported no discomfort. Besides, he experienced mild facial acne, but resolved without additional intervention after letrozole discontinuation. No other obvious adverse reactions were observed.

**Conclusion:**

The novel therapy of PEG-rhGH and letrozole demonstrated promising potential in improving NAH, while adhering to safety profiles. This case represented the first attempt to use this dual therapy for NAH enhancement in an adolescent boy with NS and a higher BA than HA.

## Background

Noonan syndrome (NS) is a congenital genetic disorder with an autosomal dominant inheritance pattern (or occasional *de novo* mutations) and a prevalence of 1 in 1,000 to 2,500 individuals (1,2). Over 20 genes are associated with NS, with PTPN11 being the first identified (2). Core features include distinctive facial characteristics, developmental delay, and short stature (a primary symptom), along with frequent multisystem abnormalities (cardiac, digestive, skeletal, urinary, endocrine, etc.) (3,4). With increasing age, the facial features of NS patients may become less typical; however, growth retardation is observed in almost all children, and in particular, those with pathogenic variants in PTPN11 may show greater impaired growth (4,5).

Growth failure in NS is attributed to growth hormone (GH) deficiency and partial GH insensitivity (6). Accordingly, recombinant human GH (rhGH) is the primary treatment, effectively improving childhood height with few serious adverse events. However, definitive conclusions regarding the effects of this therapy on near-adult height (NAH) and long-term health outcomes remain unavailable (6,7). Moreover, short-acting rhGH efficacy is further compromised poor compliance due to daily administration, particularly in adolescents. Long-acting rhGH represent a significant advancement in the treatment of pediatric GH deficiency. They have demonstrated non-inferiority to daily rhGHs, showing similar efficacy in terms of growth velocity and safety profiles (8–10). Notably, the once-weekly injection regimen has improved treatment adherence relative to the daily injection schedule. As indicated in clinical guideline, this benefit is especially valuable for populations with inherent barriers to daily injection compliance, including patients on polypharmacy, those with needle phobia or behavioral disorders, young children, adolescents, and children from families with irregular caregiving or who participate in child-focused group activities (11).

Pegylated rhGH (PEG-rhGH, Changchun GeneScience Pharmaceutical Co., Ltd., China), the first domestically developed long-acting rhGH formulation in China, was approved for marketing in the country in 2014. In comparison to traditional rhGH, PEG-rhGH enhances protein stability through the covalent modification of proteins with polyethylene glycol, which prevents non-specific adsorption and immunogenicity while extending the drug's metabolic half-life to achieve a prolonged effect (12). Currently, prospective clinical studies have demonstrated its effectiveness and safety regarding height growth in patients with idiopathic short stature (ISS) and GH deficiency (GHD) (13,14). Furthermore, for patients entering puberty, especially those with higher BA relative to HA, mitigating BA progression is critical to optimizing height. Currently, two main classes of agents are used for this purpose: gonadotropin-releasing hormone analogs (GnRHa) and aromatase inhibitors (AIs) (15,16). GnRHa suppresses the hypothalamic-pituitary-gonadal axis, to delay BA advancement but may reduce growth velocity, thereby hindering linear growth. In contrast, AIs do not exhibit such obvious drawbacks. Additionally, GnRHa may lead to the arrest or regression of sexual development. In this situation the child will not only be quite short, but also sexually infantile compared with his/her peers; the latter could have psychological implications for some children. Conversely, AIs slowed BA progression without disrupting normal pubertal development, allowing adolescents to maintain age-appropriate psychological well-being (17). Given these characteristics, AIs represent a favorable option for NS patients with advanced BA than HA who do not present with precocity or rapid pubertal progression. Notably, letrozole demonstrating a superior inhibitory effect on estrogen production, exceeding 99.1% (16).

Existing data has demonstrated that rhGH combined with letrozole effectively improves short stature in adolescent boys while maintaining a favorable safety profile (15,18). However, to our knowledge, no reports have described the treatment of PEG-rhGH and letrozole for NAH. Herein, we present the first case of novel therapy in the treatment of NAH in a NS late-puberty boy with higher BA than HA.

## Case presentation

On August 28, 2015, an 8-year-old boy was admitted to a local hospital due to an 8-year history of developmental delay. However, the etiology of the developmental delay was not evident. He was born at full-term via natural delivery, with a birth weight of 4.5 kg (birth length not recorded). The patient's father and mother had heights of 183 cm and 160 cm, respectively, with the patient's genetic target height (GTH) estimated at 178 ± 5 cm. Upon admission, the patient's height was 121.3 cm (<3rd), and his weight was 20 kg. He attended school regularly and has no intellectual disability or grammatical developmental delay. The patient's skin was smooth, with no evidence of rash or bleeding. There was no palpable enlargement of the thyroid gland, nor were there signs of pectus carinatum or pectus excavatum. No abnormalities were detected in cardiopulmonary, hepatosplenic, or extremities joint examinations. The development of secondary sexual characteristics was noted: bilateral breasts were at Tanner B1, bilateral testes measured approximately 1.5 mL, pubic hair was at Tanner I, and there was an absence of axillary hair.

Laboratory tests showed no abnormalities in routine blood, urine, liver function, renal function, blood glucose, or electrolytes. Thyroid function tests revealed: thyroid stimulating hormone (TSH) 4.520 mIU/L (Reference: 0.51–4.3 mIU/L), triiodothyronine (T3) 2.30 nmol/L (Reference: 1.4–3.34 nmol/L) andthyroxine (T4) 110.2 nmol/L (Reference: 76.1–170 nmol/L). Insulin-like growth factor-1 (IGF-1) was 71.047 ng/mL (Reference: 73–385 ng/mL). Clonidine stimulation test demonstrated a peak growth hormone (GH) level of 17.86 ng/mL at 60 min. The BA of the patient was determined to be 6.5 years (Greulich-Pyle method), with a pituitary gland height of approximately 2.5 mm. Considering the clinical features and laboratory indicators, the patient was diagnosed with ISS. The predicted adult height (PAH) calculated from BA (Greulich-Pyle method) was 173.4 cm, fell within the normal range, and the patient's family declined any medical intervention.

**Figure 1:**
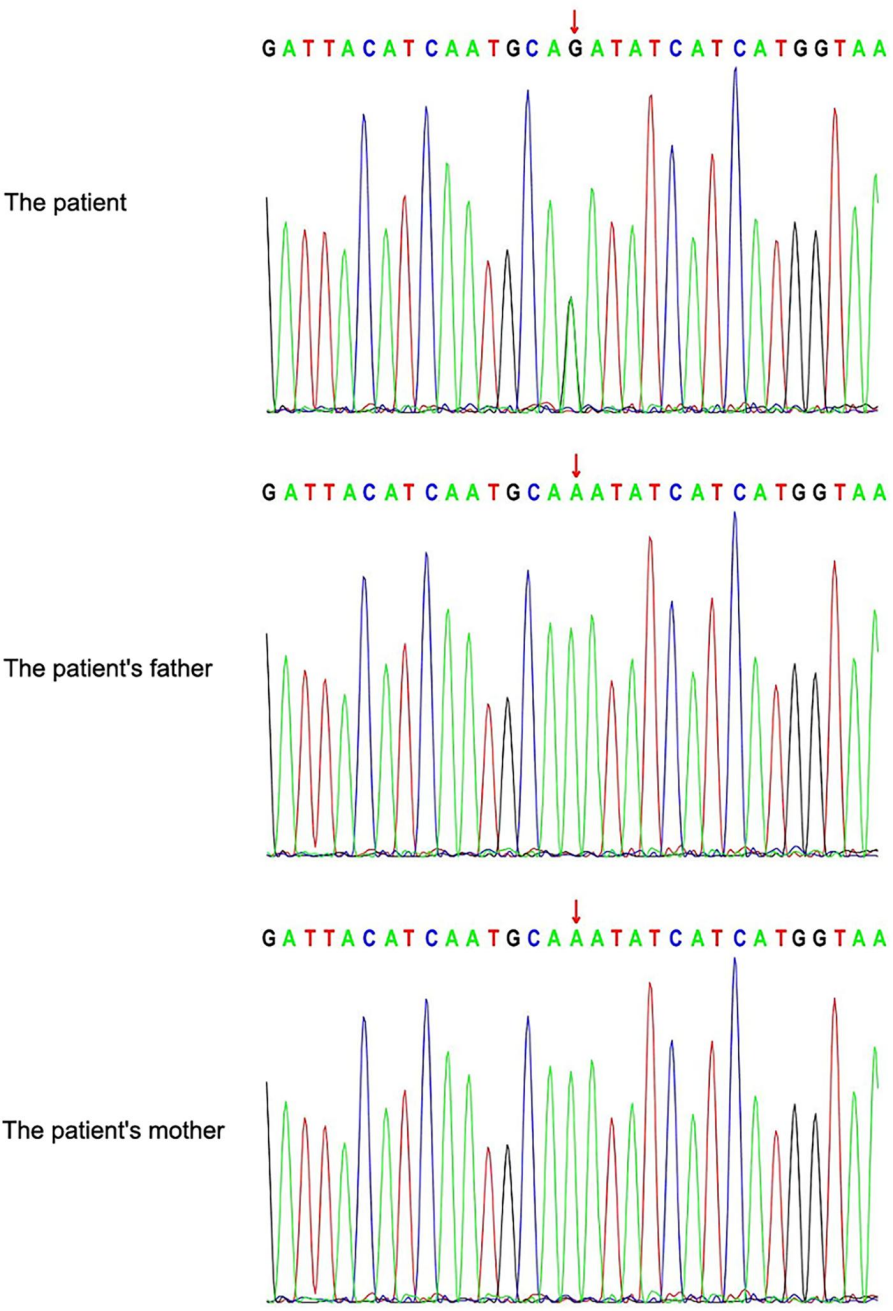
Results of whole-exome sequencing.

**Figure 2:**
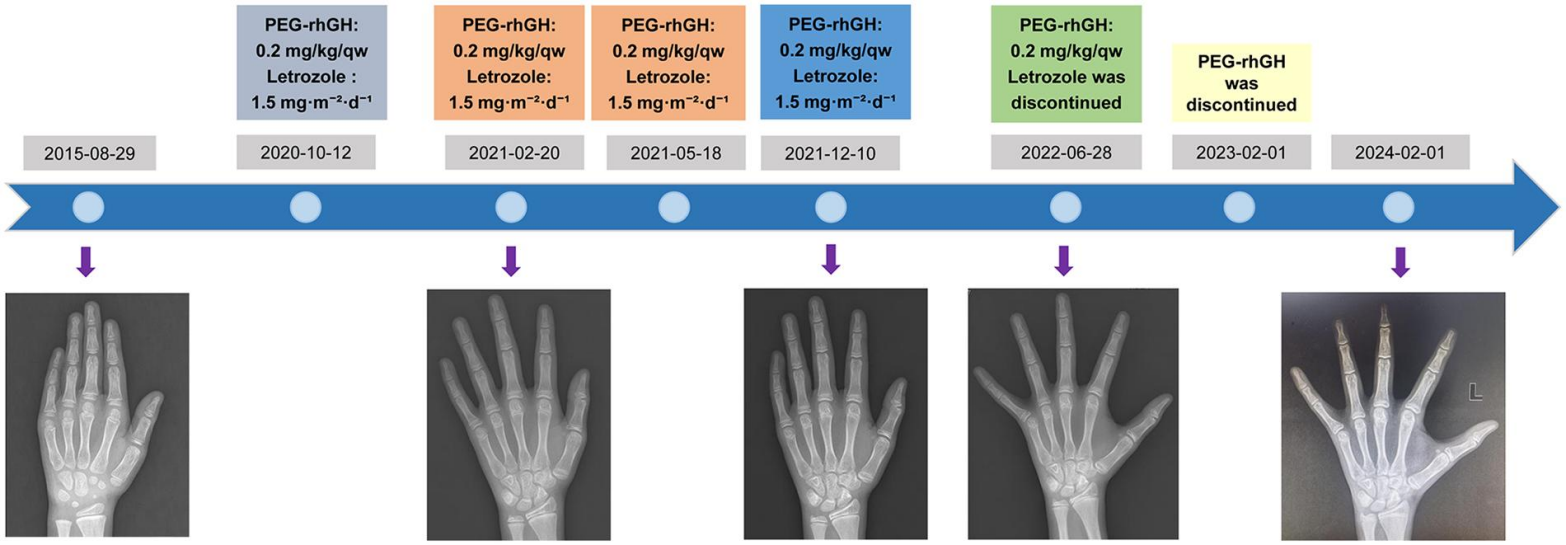
Treatment process and different periods of patient with bone age.

On October 12, 2020, the patient was admitted to our hospital due to a significant lag in height compared to peers. The height and BA of the patient were 149.5 cm (3rd) and 13.25 years, respectively. Bilateral testes measured approximately 15–20 mL and pubic hair was at Tanner II. However, BA assessment indicated that the patient's PAH (163.3 cm) was considerably below the GTH (178 cm). According to the Chinese Reference Standards for Child Growth and Development (2023 edition), his height age (HA) is approximately 11.7 years, indicating that BA was advanced relative to HA (BA-HA ≥ 1 year). Notably, we found his less typical mild ptosis and triangular face, so whole-exome sequencing was performed, which identified a well-known pathogenic variant in PTPN11 [c.922A > G (p.N308D)] (Figure 1). Based on the clinical presentation and genetic evaluation, the patient was diagnosed with NS, and both the patient and his family expressed a desire for treatment. To enhance the patient's height, following thorough discussions with the family, PEG-rhGH (subcutaneous injection, 0.2 mg/kg/qw) was administered. Concurrently, due to the higher BA than HA, letrozole (oral, 1.5 mg·m−^2^·d−^1^) was prescribed to manage BA and extend the growth period. Treatment process was showed in Figure 2.

**Figure 3:**
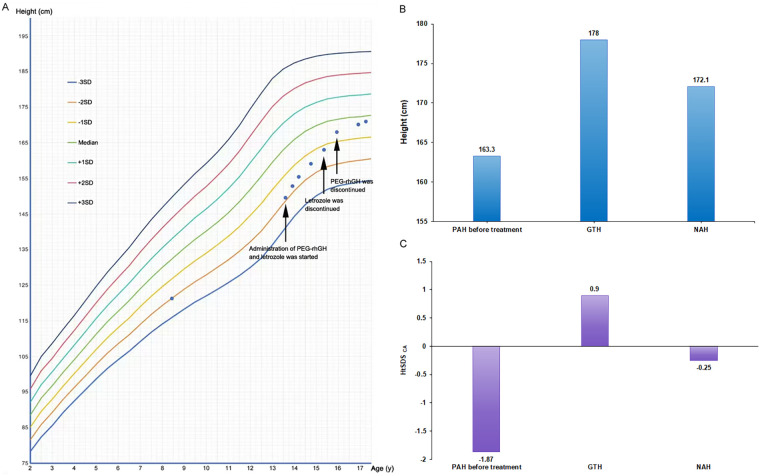
Improvement in patient height. **(A)** The patient's growth curve; **(B)** Patient height before and after treatment; **(C)** the height standard deviation score based on CA before and after treatment. PAH, predicted adult height; GTH, genetic target height; NAH, near-adult height.

During the follow-up period, the patient's height and weight were systematically monitored, and carpal bone radiographs were conducted to evaluate BA (Table 1 and Figure 2). After 2.3 years of PEG-rhGH therapy and 1.7 years of letrozole therapy, the patient's height increased by 9.7 cm in the first year, with an average growth rate of approximately 8.04 cm over the 2.3-year period. The difference between BA and chronological age (CA) decreased from −0.33 to −1.50 (Table 1). Figure 3A illustrated the growth curve of the patient. His NAH reached 172.1 cm, PAH increased by 8.8 cm. The height standard deviation score based on CA (HtSDS _CA_) improved by 1.62 (Figures 3B, C), and the HtSDS based on BA (HtSDS _BA_) improved by 1.43 (Table 1). Furthermore, there was no significant changes in thyroid function, blood glucose, uric acid, and IGF-1 levels throughout the treatment period, with all values remaining within the normal range. Similarly, his liver function, kidney function, and lipid levels also exhibited no notable abnormalities. He had only a slight elevation in testosterone level after the initial treatment, but it subsequently returned to normal, and the patient reported no discomfort. In addition, he experienced an increase in facial acne, but resolved without additional intervention after letrozole discontinuation. No other obvious adverse events (AEs) were observed during the treatment. The patients and their families were satisfied with the treatment process and the outcomes.

**Table 1:** The growth of the patient at different stages.

Time	CA (y)	BA (y)	BA-CA (y)	Height (cm)	HtSDS _CA_	HtSDS _BA_	Weight (kg)	Weight (SDS)	BMI (kg/m^2^)	Growth velocity (cm/y)	PAH (cm)
2015-08-29	8.42	6.50	−1.92	121.3	−2.04	/	20	−2.17	13.6	/	173.4
2020-10-12	13.58	13.25	−0.33	149.5	−1.87	−1.53	37	−1.65	16.6	5.5	163.3
2021-02-20	13.92	/	/	152.8	−1.69	/	45.8	−0.82	19.6	13.2	/
2021-05-18	14.17	13.5	−0.67	155.3	−1.58	−1.01	44.5	−0.95	18.4	10.0	166.6
2021-12-10	14.75	13.5	−1.25	159.2	−1.46	−0.50	51	−0.57	20.1	6.7	169.7
2022-06-28	15.33	13.75	−1.58	163	−1.19	−0.20	52.7	−0.49	19.8	7.4	171.5
2023-02-01	15.92	/	/	168	−0.56	/	52	−0.76	18.4	8.6	/
2024-02-01	16.92	15.5	−1.42	170	−0.38	−0.16	51	−1.17	17.4	2	171.5
2024-10-09	17.5	16	−1.50	171	−0.25	−0.10	55	−0.75	18.8	1.5	172.1

CA, chronological age; BA, bone age; HtSDS _CA_, height standard deviation score based on CA; HtSDS _BA_, height standard deviation score based on BA; BMI, Body mass index; PAH, predicted adult height.

## Discussion

The diagnosis of NS is predominantly clinical, although genetic defects can be detected in 80% of patients (2). Herein, we report a distinctive NS patient who presented with an atypical clinical course: at 8 years of age, the patient only manifested short stature without classic NS facial features and normal PAH, leading to missed diagnosis and unconsidered genetic testing. By 13 years old, significant height deficit relative to age-matched peers and PAH markedly below GTH prompted re-evaluation, during which subtle NS facial features (mild ptosis, triangular face) were first identified, and genetic testing confirmed NS. This case highlights that NS misdiagnosis is prone to occur in patients with few specific clinical signs, emphasizing the necessity of detailed repeated physical examinations and consideration of genetic testing for children with unexplained short stature to avoid delayed diagnosis. Its uniqueness also lies in the patient being an adolescent boy with a BA greater than the HA. Puberty-related sex hormones dualistically regulate linear growth-promoting height while accelerating epiphyseal fusion, which limit the duration of height growth (16). This poses a unique challenge for NS patients with normal pubertal onset, short stature, and a BA greater than the height age, necessitating personalized treatment strategies.

Our choice of letrozole over GnRHa for this NS patient with a higher BA relative to HA is supported by both mechanistic and clinical considerations. This aligns perfectly with our patient's clinical profile: he did not exhibit precocity or rapid pubertal progression, so the strong suppressive effect of GnRHa was unnecessary. Instead, letrozole's ability to preserve age-appropriate pubertal development and psychological well-being, while mitigating BA progression. Although anastrozole is also a highly selective AIs, letrozole exhibits a stronger aromatase inhibition rate (>99.1% vs 96.7%). Furthermore, letrozole has far more extensive clinical experience and has been more thoroughly investigated than anastrozole in children and adolescents with idiopathic short stature, pubertal disorders, and growth hormone combination therapy. This makes letrozole the optimal therapeutic choice.

Here, we pioneered the use of PEG-rhGH instead of daily rhGH in combination with letrozole for this specific patient, leveraging evidence-based benefits of the regimen while addressing the challenges posed by the patient's boarding school setting and suboptimal treatment adherence. Mauras et al. reported that adolescent boys with ISS received treated with GH combined with AI for 2 years achieved faster height gain those on GH or AI monotherapy, with an increase of approximately 1.0 (19). A retrospective study showed that GH and letrozole combination increased PAH by approximately 11 cm and a final adult height of 172.67 cm in pubertal boys with short stature, without significant side effects (17). To our delight, after 2.3 years of PEG-rhGH treatment and 1.7 years of letrozole treatment, the patient's BA minus CA was shortened, indicating that letrozole promoted a slow increase in BA and prolonged the growth period. Ultimately, the patient's NAH reached 172.1 cm, and the HtSDS_CA_ improved from −1.87 to −0.25. These results exceeded Mauras et al.'s report of 1.0 HtSDS gain in ISS with GH plus AI (19). Additionally, our findings were aligned with phase 3 (20) and Xie et al. (21) findings that PEG-rhGH outperforms daily rhGH in growth outcomes and adherence for GHD and ISS. We noticed that letrozole was discontinued before PEG-rhGH, a decision driven by two key clinical considerations: (1) achievement of primary treatment goal: after 1.7 years of combination therapy, the PAH had approached the patient's GTH range; (2) favorable risk-benefit profile: mild facial acne and minimal added benefit of continued use. Collectively, this individualized decision was aligned with pediatric NS management principles, prioritizing treatment target achievement and long-term safety.

The treatment was well tolerated by the patients, with no serious adverse reactions occurred. Studies have shown that common AEs associated with PEG-rhGH therapy include decreased thyroid function, elevated blood glucose and IGF-1 levels, transient edema, and injection site reactions (12–14). AIs may lead to increased testosterone levels and impaired fasting blood glucose levels (18). However, during the entire observation period, the patient's thyroid function, blood glucose, and IGF-1 levels remained within normal limits without significant changes. He had only a slight elevation in testosterone level after the initial treatment, but it subsequently returned to normal, and the patient reported no discomfort. In addition, he experienced a slight increase in facial acne, which was related to elevated testosterone level (22), but resolved without additional intervention after drug discontinuation.

The optimal management of these patients entails early accurate diagnosis and tailored interventions before puberty (18). In our study, post-pubertal intervention-attributed to potential misdiagnosis and limitations of early PAH estimation due to early age constraints-yielded positive outcomes; however, the therapeutic efficacy may have been underestimated. We acknowledge the inherent limitation of a single case, and future large-scale clinical trials are therefore warranted to accurately evaluate the efficacy of this novel therapy.

## Conclusion

A novel therapy involving PEG-rhGH and letrozole demonstrated a good benefit-risk profile improving NAH for adolescent NS patients with a higher BA than HA, supporting the feasibility of this therapeutic strategy.

## Data Availability

The datasets presented in this article are not readily available because of ethical and privacy restrictions. Requests to access the datasets should be directed to the corresponding author.
